# Analyzing the impact of metabolism on immune cells in tumor microenvironment to promote the development of immunotherapy

**DOI:** 10.3389/fimmu.2023.1307228

**Published:** 2024-01-08

**Authors:** Yanru Long, Houhui Shi, Yuedong He, Xiaorong Qi

**Affiliations:** Department of Gynecology and Obstetrics, Key Laboratory of Birth Defects and Related Diseases of Women and Children (Sichuan University), Ministry of Education, West China Second Hospital, Sichuan University, Chengdu, China

**Keywords:** metabolism reprogramming, tumor microenvironment, immunotherapy, glucose metabolism, lipid metabolism, amino acid metabolism

## Abstract

Tumor metabolism and tumor immunity are inextricably linked. Targeting the metabolism of tumors is a point worth studying in tumor immunotherapy. Recently, the influence of the metabolism of tumors and immune cells on the occurrence, proliferation, metastasis, and prognosis of tumors has attracted more attention. Tumor tissue forms a specific tumor microenvironment (TME). In addition to tumor cells, there are also immune cells, stromal cells, and other cells in TME. To adapt to the environment, tumor cells go through the metabolism reprogramming of various substances. The metabolism reprogramming of tumor cells may further affect the formation of the tumor microenvironment and the function of a variety of cells, especially immune cells, eventually promoting tumor development. Therefore, it is necessary to study the metabolism of tumor cells and its effects on immune cells to guide tumor immunotherapy. Inhibiting tumor metabolism may restore immune balance and promote the immune response in tumors. This article will describe glucose metabolism, lipid metabolism, amino acid metabolism, and immune cells in tumors. Besides, the impact of metabolism on the immune cells in TME is also discussed for analyzing and exploring tumor immunotherapy.

## 1 Introduction

**Figure 1:**
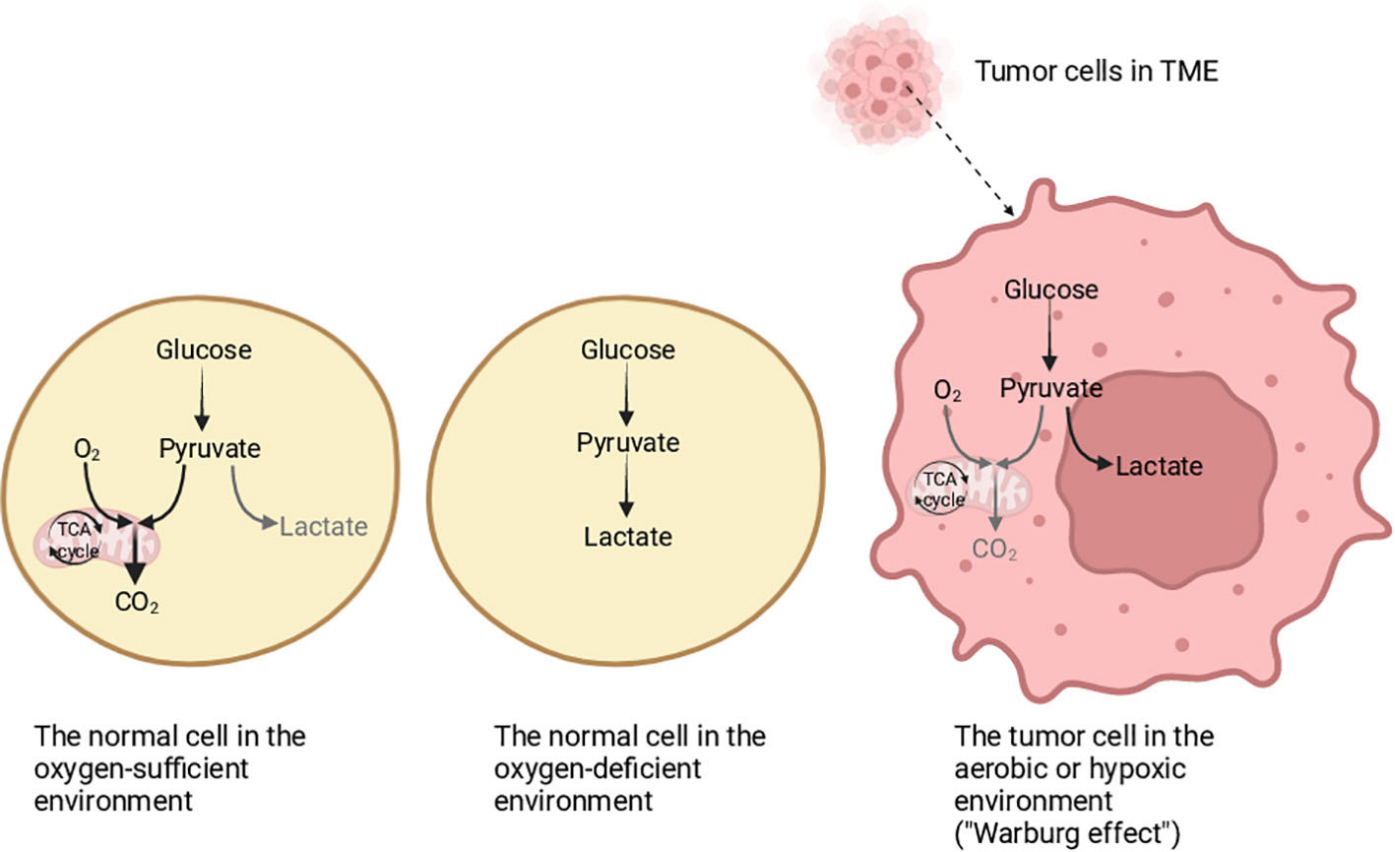
The differentiation of glucose metabolism in normal cells and tumor cells. In an oxygen-sufficient environment, the glucose metabolism in normal cells is mainly oxidative phosphorylation, and a small part is glycolysis. In an oxygen-deficient environment, the normal cells mainly obtain energy through glycolysis. In tumor cells, whether there is hypoxia or not, energy is mainly obtained through glycolysis, which is called the “Warburg effect”. Created with "BioRender.com".

Tumor metabolism has been universally studied by a lot of researchers all over the world, and the heterogeneity of cellular metabolism is one of its most worthy hallmarks to be explored (1). In normal cells, the glycolysis and tricarboxylic acid (TCA) cycle can provide energy for them, but different from normal cells, tumor cells produce lactic acid via glycolysis even if in an environment with sufficient oxygen, which is termed as “Warburg effect” (2–5) (**Figure 1**). The “Warburg effect” reflects the features of tumor cells changing their metabolism patterns to be suited to the tumor microenvironment (TME), which is known as “metabolism reprogramming”, and metabolic reprogramming is significant for the growth of tumor cells (6–8). The proliferation of tumor cells is accompanied by the collection of non-tumor cells, forming a specific environment good for tumor growth and metastasis (9). At the same time, it reshapes the TME (10). The TME affects the occurrence, progression, and metastasis of tumors greatly, and is mainly composed of cellular and noncellular portions (11). The cellular portions consist of immune cells, endothelial cells, cancer-related fibroblasts, and so on (12). The non-cellular portions contain largely the extracellular matrix, which includes glycoproteins and protein polysaccharides, to support the structure of tissue (12–14). Additionally, the portions in TME have a non-negligible impact on the immune response. Immune cells undergo metabolic changes to adapt to the TME, thus destroying the effectiveness of the immune response (15). One of the points is that the function of the immune system is affected by tumor cells, so the immune system cannot identify tumor cells normally, for example, tumor cells produce specific metabolites to inhibit the function of antitumor immune cells, and another point is that tumor cells promote immunosuppressive cells to secrete immunosuppressive molecules, thus affecting anti-tumor immunity, which is favorable to immune escape and tumor metastasis to a certain extent (8).

The mechanism of tumor metabolism affecting immune escape is being studied in depth (12). It is indicated that the inhibited immune response is in connection with a variety of factors such as the metabolic patterns of substances in the TME (16). It is gradually found that the reprogramming of metabolism in TME has a huge impact on immunotherapy for tumors, for instance, glucose metabolism, amino acid metabolism, and fatty acid metabolism can regulate immune checkpoint therapy (12). Therefore, targeting metabolism reprogramming is a tumor treatment method worth studying. It is worth mentioning that much attention has been paid to tumor cell metabolism, but little is recognized about the role of immune cells in the TME. However, the metabolism of immune cells does not only affect their function but also has an impact on the occurrence and progression of tumors.

It is indicated that the immune response in the tumor microenvironment possibly be affected when the metabolism of immune cells changes, and the metabolism of immune cells is crucial to the growth of tumors (17). Recently, immunotherapies have been regarded as a promising tumor treatment by activating immune cells or through transplantation of engineered cells for targeting and eliminating tumor cells (14). It makes sense to analyze the function of immune cells in the TME and the influence of TME on immune cells, to provide some clues for facilitating the tumor immunotherapy (17).

## 2 The metabolism of tumor cells in TME

The TME, a place for the metabolism of cells, includes extracellular matrix, tumor cells, immune cells, stromal cells, and so on (18, 19). Tumor cell metabolism and proliferation require oxygen, but slow angiogenesis can lead to hypoxia in tumor tissue (20). Most tumor tissues form an environment that lacks nutrients and oxygen, so tumor cells are necessary to alter their metabolic patterns to adapt to such an environment (21–24). For instance, it is found that hypoxia can facilitate glycolysis in tumor cells, and can also promote lipid metabolism by activating the PI3K/AKT pathway (20). That is to say, tumor cells undergo metabolic reprogramming of several substances, including glucose, lipids, and amino acids, but the most famous one is the “Warburg effect” (25–27). Metabolism reprogramming is one of the ways for tumors to avoid the attack of the immune system and inhibit the immune response, which plays a crucial role in adapting to TME for tumor cells (28). Tumor cells can inhibit the function of immune cells by competitively ingesting nutrients such as amino acids, and can also affect the function of immune cells by producing complex and variable metabolites (29).

### 2.1 Glucose metabolism

In TME, whether oxygen is sufficient or not, tumor cells make use of plenty of glucose for glycolysis to generate lactic acid (30–32). As we all know, hypoxia-inducible factor-1 (HIF-1) induced by hypoxia can determine the modes of glucose metabolism, and a variety of substances in TME increase the amount of HIF-1 protein and also promote glycolysis (30). Also, it is found that the enhancement of glycolysis in tumor cells is regulated by many factors, such as HIF-1, p53, and PI3K/AKT pathways (33, 34). And the increase of aerobic glycolysis in tumor cells produces more lactic acid. If lactic acid continues increasing, it will lead to acidity or even acidosis in the cells (33). According to research, lactic acid can be excreted into the tumor matrix through monocarboxylate transporters 4 (MCT4), and it can not only promote the increase of HIF-1 and vascular endothelial growth factor (VEGF) but also be regarded as a source providing energy for tumor cells (20). What’s more, lactic acid is dissociated into lactate and H+ in the body. The more lactic acid, the more H+ is produced. The increased H+ can reduce the pH of TME, causing tumor immunosuppression (35). Although the amount of ATP produced by monomolecular glucose through oxidative phosphorylation in mitochondria is higher than that of aerobic glycolysis, aerobic glycolysis produces ATP faster, which is beneficial for tumor cell growth (36, 37). In addition, aerobic glycolysis also reduces the demand of tumor cells for oxygen and mitochondria (20).

### 2.2 Lipid metabolism

When glucose is deficient in the TME, tumor cells and immune cells tend to perform lipid metabolism (38). In addition, immune cells can also make use of the ketone bodies produced by the fatty acids oxidation to obtain energy (20). Lipids include phospholipids, cholesterol, triglycerides, fatty acids, and many other types (39–42). Lipid metabolism includes anabolism and catabolism, and the catabolism is commonly known as β-oxidation (43).

Lipid metabolism is an important energy source, which can promote the proliferation of cells and enhance the function of cells (44). Apart from providing the energy needed for cell metabolism, lipids can also promote cell migration in other ways, for instance, lipids can form cell membranes, participate in signaling, and so on (45–48). In normal cells, lipids can be obtained by ingesting lipids outside the cell, or by synthesizing lipids in cells, and then metabolizing lipids (49, 50). The lipid metabolism in tumor cells is significantly different (51, 52). The activation of oncogenes induces lipid metabolism to provide energy for tumor cells, eventually promoting the proliferation and metastasis of tumors (53). The crazy proliferation of tumor cells needs the support of several lipids (54). Not only can tumor cells synthesize lipids by using some enzymes, including fatty acid synthase (FASN), sterol regulatory element-binding proteins (SREBPs), acetyl-coenzyme A (Ac CoA), stearoyl-CoA desaturase 1 (SCD1), acetylCoA carboxylase (ACC), but also they can absorb lipids directly from the external environment through different ways (45).

For example, low-density lipoprotein (LDL) can enter tumor cells with the help of LDL receptors. Besides, cholesterol can enter tumor cells mediated by fatty acid translocase, such as CD36 (20). In TME, because of the hypoxic environment, HIF promotes the intake and synthesis of fatty acids in tumor cells, which is beneficial for lipid accumulation (55). In addition to fatty acids, phospholipids and cholesterol are also two kinds of lipids that cannot be ignored, on the one side, cholesterol can not only form a cell membrane but also be converted into steroid hormones under certain conditions, on the other side, steroid hormones can spread freely from serum to cells (20). When steroid hormones bind to receptors in tumor tissue, tumor cells can proliferate, and this mechanism is probably related to the development of breast cancer (56). Cholesterol is a part of the cytomembrane, maintaining the structure of cells, and its metabolites can regulate the function of immune cells (57). Phospholipids are divided into glycerophospholipids and sphingolipids, which can not only form cell membranes but also participate in some signaling pathways, such as the PI3K signaling pathway, among them, phospholipids related to arachidonic acid (AA) have attracted widespread attention (58). In tumor cells, if lipids are abundant, they are probably stored in lipid droplets (LDs), and it is reported that the storage forms include triglycerides and cholesterol esters (45). Therefore, the obvious increase in the number of LDs has become one of the characteristics of tumors (20). Lipid metabolism is the basic metabolism and the energy source of tumor cells, eventually promoting the growth and development of tumors.

### 2.3 Amino acid metabolism

Apart from glucose metabolism and lipid metabolism, amino acid metabolism in TME has gradually attracted attention. Amino acids, organic compounds, are the basic units that make up proteins, and it has been shown that amino acids are related to tumor cell proliferation and metastasis (15). Although amino acids have a nonnegligible influence on the function of cells, they cannot directly enter into cells. It is demonstrated that the hydrophilic nature of amino acids determines that they cannot freely pass through the cell membrane, in fact, amino acids need to enter cells with the assistance of various transporters (17).

Besides, amino acid metabolism is reprogrammed in TME, and it has been confirmed that amino acid metabolism reprogramming includes changes in the intake rate of amino acids, changes in pathways, and abnormality of key enzymes (17). Amino acids are of significance for tumor cells, such as arginine, methionine, glutamine, and so on (59). The rapid proliferation of tumor cells requires the intake of a large number of amino acids, and the uptake of amino acids requires the assistance of transport proteins, so amino acid transport proteins can regulate the uptake rate of amino acids and influence tumor development, for example, glutamine enters tumor cells with the assistance of alanine-serine-cysteine transporter 2 (ASCT2) (17). Compared with normal cells, tumor cells need more glutamine to proliferate, so the expression of ASCT2 in tumor cells increases. In addition, enzymes and signaling pathways related to amino acid metabolism can also affect tumor proliferation, for example, liver kinase B1 (LKB1), also known as serine-threonine kinase 11 (STK11), is inhibited when combined with asparagine (17). Therefore, asparagine metabolism can affect the proliferation and metastasis of tumor cells by affecting the LKB1-AMPK signaling pathway (15).

For tumor cells, plenty of amino acids can promote their growth and development. But, as mentioned above, amino acids need amino acid transport proteins to enter cells, so tumor cells cannot ingest amino acids indefinitely. Different tumor cells may ingest different types of amino acids. Nevertheless, amino acid metabolism is also vital for promoting tumor development, which is also worth studying.

## 3 Effect of metabolism on immune cells

**Figure 2:**
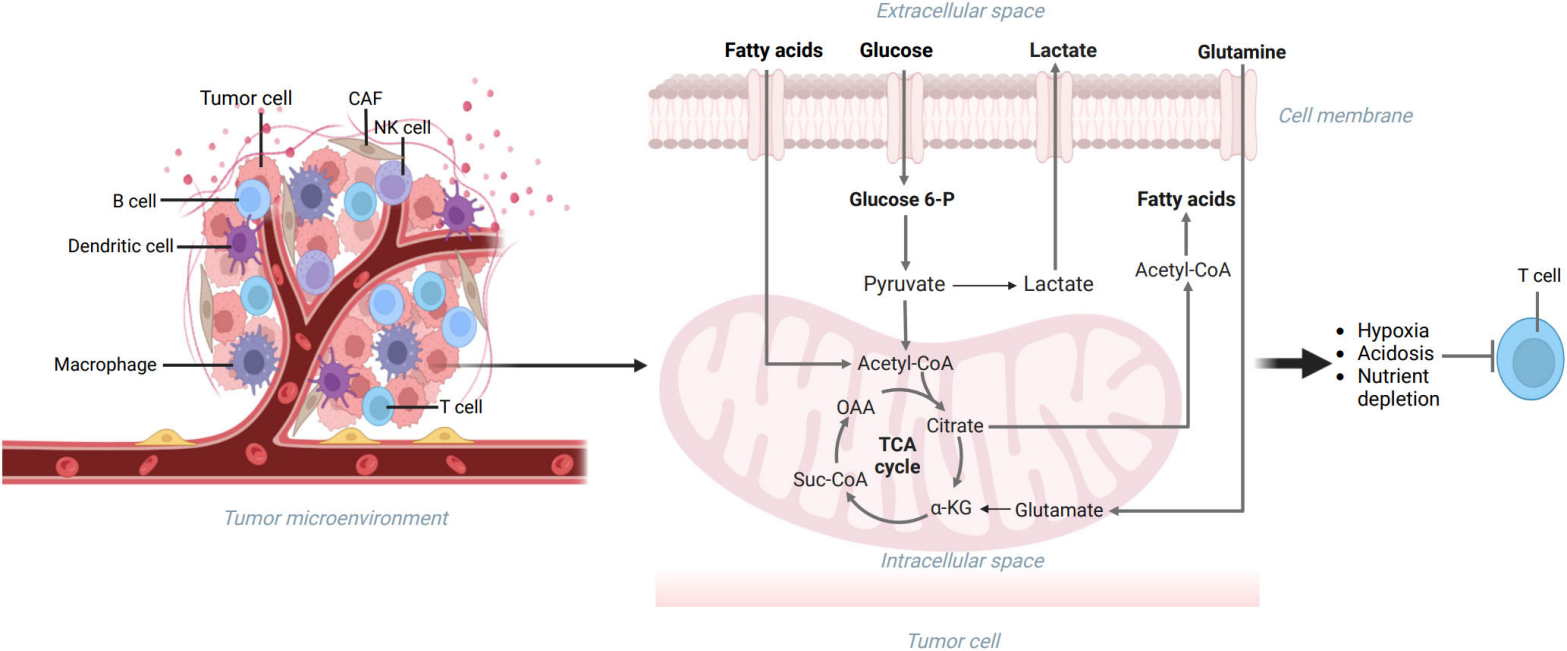
The TME includes extracellular matrix, tumor cells, and immune cells. The metabolism reprogramming of glucose, lipids, and amino acids can be seen in tumor cells, which can make the TME characterized by nutrient deficiency, lactic acid accumulation, hypoxia, and immunodeficiency. Created with "BioRender.com".

As we all know, a tumor is a kind of disease characterized by immunodeficiency, and tumor cells can escape the recognition and removal of multiple immune cells in many pathways (60). The TME, characterized by nutrient deficiency, lactic acid, lipid accumulation, and hypoxia, influences the role of the immune system and hinders the immune response (**Figure 2**) (33, 44). Additionally, more and more studies have shown that some metabolites in TME can restrain immune activities (25). To cope with the pressure of the TME, immune cells regulate their metabolic patterns to inhibit tumor development (56). Therefore, we need to study the metabolic modes of immune cells in TME and the effects of immune cells on tumor growth.

The immune system is composed of many types of cells, including neutrophils, natural killer cells, macrophages, monocytes, eosinophils, and basophils, and the immune system mainly maintains the homeostasis of the internal environment through immune response (61). Usually, some pathological conditions, such as tumors and inflammation, may influence the immune cell metabolism (60). Tumor-infiltrated immune cells are dual, some of which has the effect of inhibiting tumor proliferation and metastasis, such as effector CD8+T (Teff) cells, memory CD8+T (Tmem) cells, natural killer cells (NK cells), B cells, M1 macrophages, dendritic cells (DCs), N1 neutrophils and so on, while others may be of benefit to the development of tumors, including Treg cells, M2 macrophages, myeloid-derived suppressor cells (MDSCs), and N2 neutrophils (62). Among them, cytotoxic T cells, T effector cells, dendritic cells, and B cells mainly remove cancer cells by recognizing specific antigens, while natural killer cells and macrophages block tumor development through non-specific immune responses (63). Although these cells can prevent tumor development to a certain extent, tumors gradually adapt and promote the role of cells suppressing the immune response, such as tumor-associated macrophages and regulatory T cells, and this feature of tumors forms an immunosuppressive microenvironment, which facilitates ultimately tumor proliferation and metastasis (64).

Generally speaking, immune cells can recognize and inhibit tumor cell proliferation, and even destroy tumor cells (20). However, in TME, the metabolism reprogramming of tumor cells can escape immune surveillance and influence the metabolism of immune cells to promote tumor development (52, 65).

The glucose metabolism reprogramming is beneficial for facilitating the proliferation and development of tumors, and it also has an influence on immune cells in TME (32). The proliferation of tumor cells and immune cells depends on glycolysis, tumor cells use glucose competitively, which makes immune cells lack nutrients and unable to perform their functions (66). In addition, metabolites produced by tumor cells, such as lactic acid, can also inhibit the role of immune cells, eventually weakening anti-tumor immunity and promoting tumor immune escape (67). It is understood that enzymes in the process of glycolysis may also affect the function of immune cells, among them, hexokinase 2 (HK2) and phosphofructokinase 1 (PFK1) respectively participate in the transformation of glucose into glucose 6phosphate and glucose 6-phosphate into fructose 6-phosphate (68). Besides, lactate dehydrogenase A (LDHA) and pyruvate kinase M2 (PKM2) participate in the process from phosphoenolpyruvate to pyruvate and then to lactic acid (32). Interestingly, HK2 is inseparable from the degree of infiltration of immune cells. PFK1 affects the differentiation of macrophages. PKM2 can promote oxidative phosphorylation and the “Warburg effect”, in addition, LDHA can facilitate the production of lactic acid and affect immune cells, eventually leading to immunosuppression (32).

In TME, the variation of metabolic modes of tumor cells inhibits the immune response and promotes tumor development by influencing the composition and function of infiltrated immune cells (69). When glucose is deficient in TME, tumor-infiltrated immune cells may change their metabolic patterns, that is, to maintain their functions by ingesting and metabolizing fatty acids (22). It is found that immune cells that promote the development of tumors generally obtain energy through lipid metabolism to maintain their functions (20). However, it has been proved that excessive lipids may inhibit immune cells and impair the anti-tumor immune response (70). Accordingly, lipid metabolism in TME may impact the survival, progression, and function of immune cells (39). And now, the influence of lipid metabolism on the function of immune cells has been widely discussed.

Amino acid metabolism in TME can also have an effect on immune cells, which is characterized by complexity and diversity (5). For immune cells, amino acids can promote the synthesis of proteins, which are beneficial for immune cells. Not only can amino acid metabolism provide energy for immune cells, but the crucial enzymes in amino acid metabolism can influence immune cells (59). Nevertheless, as mentioned above, amino acids need transport proteins to enter into cells, so immune cells cannot ingest amino acids indefinitely. And different immune cells ingest different types of amino acids (15). Disappointingly, amino acid metabolism reprogramming in TME is harmful to immune cells, on the one hand, the increase of amino acid transporters on tumor cells can promote the entry of amino acids into tumor cells, while the number of amino acids entering immune cells decreases, on the other hand, once the amino acids metabolism products in tumor cells are released, they can damage immune cells in TME (17). Therefore, more and more attention has been paid to analyzing the impact of amino acid metabolism on immune cells.

### 3.1 Tumor-infiltrating T cells

T cells originate from the thymus and enter the cycle after completing differentiation, the survival and proliferation of these T cells primarily depend on oxidative phosphorylation and the oxidation of fatty acids (71). T cells have various types, including CD4+ cell groups and CD8+ cell groups. CD4+ cell groups include regulatory T cells (Treg), follicular helper T cells (Tfh), T helper cells 1 (Th1), T helper cells 2 (Th2), T helper cells 17 (Th17), while CD8+ cell groups include naive T cells, memory T cells (Tmem), effector T cells (Teff), activated cells, chronic activated T cells (56). Generally speaking, T cells trigger a variety of immune responses by recognizing antigens to maintain the balance of the immune system. In different TME, T cells influence tumor proliferation and tumor development by modulating the anti-tumor immune response (56). There are also differences in the effects of different T cells on tumors. Teff cells can damage tumor cells by producing cytokines, and this activity needs to increase the uptake of glucose and speed up glucose metabolism, while Tmem cells can exist in tumor tissue for a relatively long time, so they can help control tumor growth (20). Although Th17 cells inhibit anti-tumor immune response and promotes tumor proliferation, it also aggregates immune cells (56). Although Th2 cells may promote tumor development through immunosuppression, the subtype closest to immunosuppression in CD4+T cells is Treg (22). It is shown that Treg cells not only secrete inhibitory factors such as interleukin-4 (IL-4) and interleukin-10 (IL-10) but also express inhibitory molecules such as cytotoxic T-lymphocyte-associated protein 4 (CTLA-4) and programmed cell death protein 1 (PD-1) (20). Therefore, in TME, Treg cells inhibit the function of anti-tumor immune cells and ultimately inhibit the anti-tumor immune response, which is beneficial for the progress of tumors (71).

In TME, different types of T cells probably obtain energy in different metabolic pathways. CD8+ T cells are one of the essential immune cells in anti-tumor immunity(48). After being activated, CD8+ T cells are mainly transformed into effector T cells and memory T cells, and their metabolic pathways are completely different. The metabolic pathways in Teff mainly include glycolysis and glutamine metabolism, while in Tmem include oxidative phosphorylation and fatty acid oxidation (71). Apparently, effector T cells increase the uptake of glucose and speed up glucose metabolism (33).

**Figure 3:**
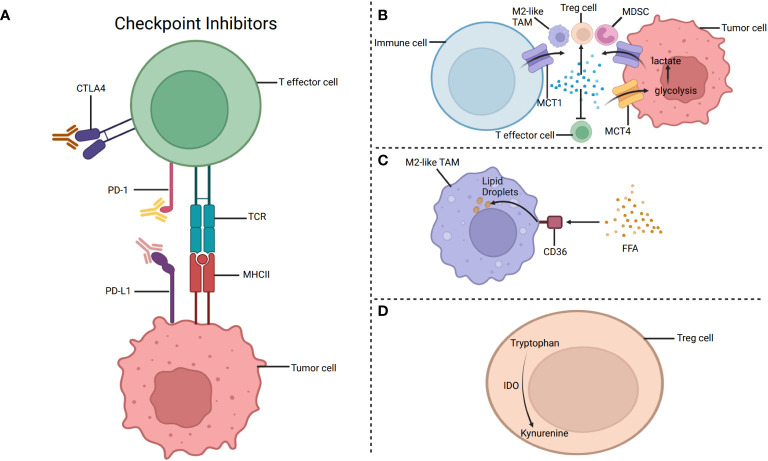
The metabolism targets for immunotherapy. **(A)** Targeting PD-1 and CTLA-4 to restore the immune function of T cells. **(B)** The lactic acid generated by glycolysis in tumor cells is transported outside the tumor cells through monocarboxylate transporter 1 (MCT1). Besides, lactic acid accumulation may inhibit the function of T effector cells and promote the function of M2-like TAMs, Treg cells, and MDSC. **(C)** CD36 is a glycoprotein that can help fatty acids pass through the cell membrane. Increased expression of CD36 enhances the fatty acid oxidation in M2like TAMs, eventually leading to immunosuppression. Therefore, CD36 inhibitors can hinder the intake of fatty acids by M2-like TAMs and ultimately achieve the purpose of inhibiting tumor progression. **(D)** IDO, produced by Tregs, decomposes tryptophan to specific metabolites, inhibiting the function of immune cells, such as effector T cells. Targeting IDO can inhibit tumor development by restoring tumor immunity. Created with "BioRender.com".

Besides, it is confirmed that T helper cells (Th cells) mainly depend on glycolysis, while Treg cells primarily depend on fatty acid oxidation (56). However, it is guessed that the glucose metabolism of tumor cells affects the metabolism and role of many T cells. Because of the competitive use of glucose by tumor cells, the amount of glucose that the glycolysis-dependent cells can use is significantly reduced (34). Hence, the TME that lacks oxygen and glucose may inhibit the development and function of the glycolysis-dependent cells (71). Nevertheless, the function of some cells that mainly rely on lipid metabolism like Treg cells is not inhibited by the TME that lacks glucose and is full of lactic acid. These cells may also inhibit the immune response and facilitate immune escape (2). Besides, increased lactic acid has been proven to hinder immunity and facilitate tumor development. The lactic acid generated by glycolysis in tumor cells is transported outside the tumor cells through monocarboxylate transporter 1 (MCT1)/monocarboxylate transporter 1 (MCT4), thus forming an acidic TME, which may eventually inhibit the metabolism of cytotoxic T lymphocytes (72) (**Figure 3**). Furthermore, as a key factor in promoting Th1 differentiation, interferon-γ (IFN-γ) changes its property in an acidic microenvironment, inducing Th1 to differentiate into Th2 and ultimately promoting tumor development (73). For example, glycolysis-induced infiltration of Th2 cells affects the treatment and prognosis of lung adenocarcinoma (73). Additionally, the increase of lactic acid in the TME can facilitate the intake of more lactic acid by Treg cells. And it is favorable for the immunosuppressive function of Treg cells. Hence, reducing the content of lactic acid in the TME or limiting the intake of lactic acid by Treg cells may do good for immunotherapy (72).

In addition to glucose metabolism, lipid metabolism can also influence the survival and function of T cells. In a nutrient-deficient TME, lipid metabolism provides T cells with the energy they need to survive. The oxidation of fatty acids affects the differentiation of Tmem cells (56). Tmem cells store lipids mainly by synthesizing fatty acids rather than ingesting fatty acids, and Treg cells seem to be similar to Tmem cells at this point. Besides, Teff cells also maintain their functions through lipid catabolization. Additionally, the content of fatty acids also affects the differentiation of Th17 and Treg cells. It is confirmed that when the content of fatty acids in TME is low, exogenous fatty acids impact the function of Th17 and Treg cells. Apart from fatty acids, other lipids, such as cholesterol and phospholipids, can impact the survival and role of T cells (56). If the intake and synthesis of cholesterol are insufficient or the discharge of cholesterol is increased, the proliferation of T cells is suppressed (56). Additionally, the cholesterol in TME hinders the immune response, causing the depletion of CD8+T cells and eventually promoting the growth of tumor cells (74). In fact, the increase of cholesterol in TME facilitates the expression of inhibitory molecules, such as PD-1, affecting the function of CD8+T cells. However, when the content of cholesterol on the cell membrane increases, such as by inhibiting recombinant acetyl coenzyme acetyltransferase 1 (ACAT1), the function of CD8+T cells is promoted (56).

One of the necessary conditions for the proliferation of T cells is to ingest amino acids, so amino acid metabolism can influence the proliferation and role of T cells (15). In TME, the metabolic rate of tumor cells is many times higher than that of normal cells. Tumor cells competitively inhibit immune cells from ingesting amino acids, for example, the increased intake of glutamate by tumor cells reduces the intake of glutamate by T cells. So the proliferation and activation of T cells are hindered, especially effector T cells. Besides, the lack of tryptophan or cysteine inhibits the proliferation of T cells and affects the role of T cells (75). Moreover, CD8+T cells lose the expression of the mechanistic target of rapamycin (mTOR) due to the deficiency of RagD-dependent amino acids in TME. It has been proven to reduce the growth of T cells and inhibit the anti-tumor immune response (16). Some studies have proved that supplementing amino acids can restore the function of T cells to a certain extent (17).

### 3.2 Tumor-associated macrophages

Macrophages, differentiated from monocytes, are widely found in organisms. It is known that macrophages can not only kill pathogens and maintain tissue homeostasis but also participate in immune regulation (45). Macrophages differentiate into M1 macrophages and M2 macrophages in the face of environmental changes (76). Macrophages can be aggregated by some cytokines and chemokines in the TME, including C-C motif chemokine ligand 2 (CCL2), vascular endothelial growth factor (VEGF), and macrophage colony-stimulating factor (M-CSF) (77). Macrophages in tumor tissue are referred to as tumor-associated macrophages (TAMs) (45). It is understood that TAMs are a class of immune cells that account for a large proportion of TME, and they are connected with tumor growth and tumor development (22). TAMs, as antigen-presenting cells, are heterogeneous cell groups in TME, which can transform from a static state to an activated state through the process of activation (78, 79). In TME, TAMs gather in hypoxic areas and quickly undergo glycolysis. In addition, TAMs can also secrete immunosuppressive cytokines, and vascular endothelial growth factors to promote tumor development (80). On the one hand, TAMs can promote tumor replication and invasion through colony-stimulating factor 1 and epidermal growth factor, on the other hand, they can express immunosuppressive factors like interleukin to facilitate the immune escape of tumor cells (81).

TAMs include M1-like macrophages and M2-like macrophages, and there are differences in their energy sources and metabolic patterns (45). Specifically, the energy source of M1-like macrophages is primarily glycolysis, while the energy of M2-like macrophages is mainly provided by fatty acid oxidation and oxidative phosphorylation (80). Interestingly, different types of macrophages have different metabolic patterns. In glucose metabolism, the expression of enzymes and factors connected with glycolysis in M1-like macrophages can be upregulated by HIF-1. Apart from this, IFN-γ can accelerate the consumption of glucose in M1-like macrophages and increase glycolysis. On the contrary, the glucose metabolism mode in M2-like macrophages is no longer glycolysis but is good for mitochondrial respiration and oxidative phosphorylation (45). In lipid metabolism, while the fatty acids oxidation is adjusted downward in M1-like macrophages, it can be upregulated in M2-like macrophages induced by IL-4. Besides, the metabolic characteristics of arachidonic acid are different in these two types of macrophages (80). In M2-like macrophages, the content of cyclooxygenase 2 (COX2) is higher, and the content of cyclooxygenase 1 (COX1) is lower. And it is in sharp contrast to M1-like macrophages. In amino acid metabolism, L-arginine is converted into nitric oxide (NO) and L-citrulline to promote urea circulation in M1-like macrophages, while it is converted into L-ornithine in M2-like macrophages (61).

Additionally, glutamine is related to the function of TAMs. Of course, the pathways associated with glutamine also participate in the polarization of TAMs (61). Furthermore, different types of TAMs play different functions in affecting the development of tumors (45). M1-like macrophages can produce tumor necrosis factors, inflammatory cytokines, and reactive oxygen species, which can promote inflammatory reactions and eliminate tumor cells, while M2-like macrophages produce different cytokines from M1-like macrophages, such as tumor-stimulating cytokines, which can promote tumor angiogenesis and tumor proliferation, leading to immunosuppression (82). In addition, M2-like macrophages can also produce a variety of chemokines to affect other immune cells (83).

Although TAMs can be found at all periods of tumor development, some studies have reported that M1-like macrophages are activated earlier than M2-like macrophages (Y. 45). Besides, the stimulation in TME can change TAMs from the type of pro-inflammatory to the type of anti-inflammatory, that is, from M1-like macrophages to M2-like macrophages (78, 79). More and more studies show that TAMs mostly express M2 phenotypes in TME. This feature can create an immunosuppressive microenvironment to affect tumor proliferation and metastasis (84).

Glucose metabolism, lipid metabolism, and amino acid metabolism in TME affect many aspects of TAMs. Lactic acid is a product of glycolysis in TME. Tumor cells can activate TAMs by producing lactic acid to stimulate immune escape (29). Lactic acid can also promote the glycolysis mode in M2-like macrophages, that is, oxidative phosphorylation (61). It indicates that key enzymes in glycolysis affect the polarization of TAMs. And it is suggested that targeting the activity of hexokinase (HK) can affect tumor development by affecting the polarization of macrophages (37). Besides, the reprogramming of glucose metabolism in macrophages also has an undesirable impact on their polarization. For instance, in TME, the glucose metabolism of TAMs has changed from oxidative phosphorylation to aerobic glycolysis, thus promoting the activation of M1-like TAM (61). Furthermore, lactic acid can facilitate the polarization of M2-like macrophages through many kinds of signaling pathways, including MCT1 and HIF-1, eventually promoting tumor development (80). In lipid metabolism, the metabolic mode of TAMs depends on the stage of the tumors and the requirements in the special environment (61). It is confirmed that some metabolites produced by the lipid metabolism of tumors affect the polarisation of TAMs (85). When the content of lipids in TAMs increases, TAMs polarize to M2-like macrophages, which eventually inhibit anti-tumor immune response and promote tumor development (76). M2-like macrophages increase the expression of arginase 1 (Arg1) and promote arginine metabolism, finally inhibiting immunity and promoting tumor development (45). Moreover, glutamine (Gln) metabolism is also vital for keeping the M2 phenotype of TAMs. Accordingly, it is demonstrated that Gln metabolism is a promising target (5). It can be seen that the function of TAMs and the influence of TME on TAMs are diverse and complex. In addition to the above, there are many aspects of TAMs and the impacts of TME on TAMs that deserve more attention and further research.

### 3.3 Natural killer cells

Natural killer cells (NK), a subgroup of inherent lymphocytes, have strong anti-infection and anti-tumor ability, and can directly remove tumor cells by exerting cytotoxic activity (86). NK cells are elements of the innate immune response, which can aggregate other kinds of immune cells after being activated in TME (22). The function of NK cells is mainly regulated by inhibitory receptors, which hinder NK cells from exerting cytotoxic activity and help tumor cells escape from immunity (86). Apart from typical inhibitory receptors, checkpoint inhibitor receptors can also be expressed on NK cells (86). Additionally, interleukin-like cytokine stimulation can modulate the metabolism of NK cells through the mTOR signaling pathway (60). By upregulating the mTOR complex 1 (mTORC1) signaling, NK cells ingest a large amount of glucose and carry out aerobic glycolysis, which promotes the function of NK cells (22). It has shown that sterol regulatory element binding proteins (SREBPs) affect lipid metabolism through the regulation of mTORC1, but further research is needed to analyze the influence of SREBPs on lipid metabolism in NK cells (60).

The metabolism of various substances in TME affects the survival, proliferation, and role of NK cells. When the content of glucose in TME is low because of the increased glucose metabolism in tumor cells, the glycolysis induced by the mTORC1 signal is inhibited, thus inhibiting the anti-tumor function of NK cells (20). Besides, lactic acid can limit the function of NK cells by inhibiting the nuclear factor of activating T cells (NFAT) in NK cells, thus promoting immune escape (72). It is confirmed that if the content of lipids in NK cells increases, the function of NK cells will be inhibited, eventually leading to immune escape (39). For example, in the TME with a large number of fatty acids, NK cells absorb exogenous fatty acids, causing the suppression of glycolysis and weakening the function of NK cells (22). Apart from fatty acids, other lipids or regulatory factors may also affect NK cells. Prostaglandin E2 (PGE2) can affect the survival and growth of NK cells, and can inhibit the function of NK cells. In addition, adiponectin secreted by adipose tissue may also hinder NK cells by affecting fatty acid metabolism (60). What’s more, the signaling pathways cannot be ignored. Signaling pathways connected with lipid metabolism in NK cells include mTOR and SREBPs (85). Some studies found that when NK cells are constantly exposed to IL-15, IL-15 may restrain the metabolism and function of NK cells through the mTOR signaling pathway (60). Therefore, it is speculated that the inhibitor of mTOR, rapamycin, may restore the function of NK cells under certain conditions (60). At present, although some studies have explored the relationship between NK cells and substance metabolism, there are not enough pieces of evidence. Apparently, it is necessary to carefully analyze the influence of substance metabolism on NK cells in TME to obtain more useful information.

### 3.4 Dendritic cells

Dendritic cells include the classical DC1 (cDC1), the heterogeneous group the classical DC1 (cDC2), the plasmacytoid DC (pDC), and other subgroups, which are indispensable in TME (87). Dendritic cells, responsible for presenting antigens, strengthen the anti-tumor effect by activating T cells. The classical DC1 can ingest and submit antigens to T cells, for instance, submitting antigens to MHC class I molecules on CD8+ T cells. The classical DC2 is believed to come from monocytes, which are divided into DC2 and DC3. They can submit the tumor antigen to CD4+ T cells. The plasmacytoid DC can be found frequently in tumors, which is connected with adverse outcomes and low survival rates (88). Some findings suggest that pDCs can promote Treg cells to secrete IL-10 to inhibit the immune response (86). The TME has a certain influence on dendritic cells. In addition to reducing the number of cDC1, tumor immune escape can also be achieved by directly inhibiting the function of cDC1 (86). Surprisingly, when the content of lipids in DC increases, the function of DC to present tumor-related antigens is weakened, causing the suppression of anti-tumor immunity (39). However, because the theory of how lipids inhibit the function of DCs has not been thoroughly studied, the way to restore the ability of DCs to present antigens may be found after in-depth research to restore anti-tumor immunity (22). At this stage, there is not much research on DCs. The function of DCs in TME and the mechanism of TME affecting DCs are not comprehensive. As a consequence, more research is needed to provide more information.

### 3.5 Other types of immune cells

Neutrophils, the main components of immune cells in the body, mainly obtain energy through glycolysis (22). They seem to promote the development of tumors in some cases. For example, when the content of glucose in TME is low, neutrophils obtain energy through fatty acid oxidation, thus inhibiting the immune response and promoting tumor growth (22). Neutrophils include N1 neutrophils and N2 neutrophils. N1 neutrophils mainly obtain energy through glycolysis to achieve anti-tumor function, while N2 neutrophils continue to ingest lipids, allowing lipids to accumulate in them and eventually prolonging their survival time (22). Besides, N2 neutrophils also inhibit the proliferation of some immune cells, such as NK cells, by secreting many kinds of enzymes, and ultimately facilitating the growth of tumors (22). MDSCs are also an important class of immune cells, which primarily depend on fatty acid oxidation to obtain energy to perform their immunosuppressive function (20). It is shown that TME has a large amount of lipids, which can promote MDSCs to ingest lipids. Therefore, MDSCs can play an antitumor function in TME, but when there is too much lipid, the function of MDSCs may be inhibited (89). There are also a variety of immune cells or other kinds of cells, playing various roles in the TME, which are affected by different factors in TME, too. For example, cancerassociated fibroblasts (CAFs) are vital stromal cells in TME, which are believed to induce epithelial-mesenchymal transition (EMT) and secrete immunosuppressive factors (40). CAFs can produce lactic acid through aerobic glycolysis, and then tumor cells ingest and use lactic acid, this is defined as the reverse “Warburg effect” (20). It is proved that CAFs may help lipids be transported to tumor cells to facilitate tumor proliferation and metastasis (22). The current research has a limited understanding of cells in TME, and some cells have not even been found. Therefore, more time is needed to understand and analyze the cells in the TME.

## 4 Targeting metabolism to explore and promote tumor immunotherapy

The methods for treating tumors include radiotherapy, chemotherapy, and targeted treatment. Regarding exploring how to treat tumors, people are committed to developing effective strategies without significantly damaging surrounding tissues and cells (22). In recent years, immunotherapy has been gradually studied and has become one of the most promising treatment methods today, and it is possible to obtain better therapeutic effects and lead to fewer side effects (20).

### 4.1 Some well-known immunotherapies related to immune cells

With the discovery that the immune system inhibits tumor proliferation, the immunotherapy of tumors has been gradually paid attention to and studied deeply (34). It is confirmed that tumors can inhibit anti-tumor immunity by establishing immune checkpoints, which is in favor of tumor immune escape (89). Immune checkpoint molecule CD28, cytotoxic T lymphocyte-associated protein-4 can lead to the depletion of immune cells, eventually promoting immunosuppression (34). At present, Immune checkpoint blockers (ICB) and adoptive-cell transfer (ACT) are important ways for immunotherapy that are related to immune cells (57). It has proved that targeting PD-1 and CTLA-4 may restore the immune function of T cells (**Figure 3**) (34). Notably, NK cells can also express PD-1, while the expression of PD-1 ligand (PD-L1) limits the delivery of antigens to CD8+T cells by DCs, so the anti-tumor immune response can be promoted by hindering the PD-1/PDL1 axis (34). Besides, blocking CTLA-4 can increase the infiltration of NK cells in the TME and indirectly enhance the antitumor effect of NK cells by depleting Treg cells. Moreover, it is found that lymphocyte activation gene 3 (LAG-3) is a promising method to treat tumors by blocking checkpoints (86). Strategies for T cell immune receptor with immunoglobulin and ITIIM domain (TIGIT) pathways and its associated receptors and ligands can provide new ideas for tumor treatment (86). Hopefully, it may be a promising strategy to combine metabolic regulators and ICB to promote the function of immune cells and augment immunotherapy (32). Additionally, ACT, such as using T cells amplified *in vitro*, enhances the ability of anti-tumor immunity by modifying immune cells, and one of the advantages of ACT is that the process of modifying immune cells does not affect other surrounding cells (34). However, ICB activates immune cells in tumors by modifying inhibitory signals, so it is limited by the TME (34).

### 4.2 Therapies worthy of further research

The influence of tumor cell metabolism on immune cells is gradually regarded as a crucial mechanism for tumor immune escape. It includes the competition between tumor cells and immune cells for substances in the TME, and the accumulation of metabolites produced by tumor cells in the microenvironment (2). Therefore, the characteristics of tumor cell metabolism provide a basis for finding therapeutic targets (34). Besides, the regulation of cell metabolism may be an important idea for tumor immunotherapy (2). Furthermore, it is confirmed that metabolism reprogramming of tumor cells forms an immunosuppressive TME, resulting in less effective tumor immunotherapy than expected (34). Therefore, analyzing the metabolism of tumor cells and the factors that inhibit the function of immune cells may enhance immunotherapy (20, 34). Since the 20th century, it has begun to study the connection between tumor cells and immune cells to discover more treatments and improve the effect of immunotherapy (39).

#### 4.2.1 Therapies related to glucose metabolism

It has shown that the transporters, enzymes, and metabolites associated with glycolysis are not negligible to the drug resistance of tumors (72). Targeting the transport proteins and enzymes needed in the process of glycolysis in tumor cells may be a new idea for immunotherapy (80). The glycolysis inhibitors can prevent the growth of tumor cells and augment the therapeutic effect, which is a promising treatment method (30). Glucose transporters(GLUTs), with increased expression in a variety of tumors, are a crucial part of metabolism in tumor cells (90). The combination of glucose transporters inhibitors and other tumor treatment methods can promote tumor treatment and improve prognosis (34).

Key enzymes in glycolysis can be used as targets for tumor immunotherapy, for example, the inhibitor of PKM2, Shikonin, can inhibit glycolysis and block tumor cells from obtaining energy through glycolysis (32). The combination of PD-1 and PD-L1 on tumor cells inhibits the function of T cells in TME and promotes immune escape. It is demonstrated that both HK2 and PKM2 are connected with the expression of PD-L1, and the treatment targeting PKM2 combined with anti-PD-1 antibodies may be a hopeful treatment for tumors (32). Phosphoglycerate kinase 1 (PGK1), one of the enzymes in the glycolysis process, can promote the progress of breast cancer and have an impact on the prognosis of breast cancer patients (62). Therefore, PGK1 may also be worth an in-depth study of the therapeutic target.

The mammalian target of rapamycin (mTOR) is a serine/threonine protein kinase that turns into complexes with other molecules in tumor cells, including mTOR complex 1 (mTORC1) and mTOR complex 2 (mTORC2), and both of them can affect the function of immune cells and promote the growth of tumor cells (17). The activation of mTORC1 can promote the expression of some glucose transporters and glycolytic enzymes, ultimately promoting tumor cells to ingest glucose and stimulating the proliferation of tumors (72). Therefore, inhibiting the mTORC1 may be regarded as one of the targets of tumor treatment (56). However, it has been confirmed that mTOR inhibitors affect the metabolism of T cells, such as Teff cells, leading to inhibit immune response (34). It is necessary to explore how to better inhibit the PI3K/AKT/mTOR signaling pathway to maximize the efficacy of immunotherapy.

CD147 is highly expressed on the surface of tumor cells. It is proved that CD147 can promote the release of vascular endothelial growth factor and extracellular matrix metalloproteinase, which is closely related to the poor prognosis of tumors (2). Besides, it is shown that CD147 can promote glycolysis in tumor cells by assisting the transport of monocarboxylates (2). What’s more, its monoclonal antibody (Licartin) can prevent the recrudesce and metastasis of liver cancer to a certain extent. Moreover, compared with the use of antiCD147 antibodies alone, the combination of multiple immunotherapy methods may achieve better anti-tumor effect (2). It is said that long noncoding RNA (lncRNA) can promote glycolysis in tumor cells and regulate some oncogenic signaling pathways, for example, TAMs enhance glycolysis of tumor cells by producing extracellular vesicles (EVs) to transfer lncRNA (91). It has been proved that targeting lncRNA in TAMs can inhibit glycolysis of tumor cells (30). Therefore, lncRNA can be used as one of the targets of tumor treatment providing a new idea (30).

#### 4.2.2 Therapies related to lipid metabolism

The lipids in TME affect the proliferation and metastasis of tumors, targeting lipid production and ingestion in tumor cells can be regarded as an anti-tumor strategy (85). Lipids are related to the metabolism of tumor cells and the function of various immune cells. Therefore, studying the characteristics of lipids metabolism in the TME and various cells helps explore new ways to treat tumors.

The increase of free fatty acids in tumor cells promotes tumor metastasis (92). However, the metastasis of tumor cells is limited by saturated fatty acids and unsaturated fatty acids, if the content of unsaturated fatty acids in cell membrane phospholipids is low, the fluidity of the cell membrane will be reduced, eventually leading to a slowdown in the invasion of tumor cells (22). Therefore, maintaining the balance of saturated fatty acids and unsaturated fatty acids is of great importance for tumor metastasis. In cholesterol metabolism, low-density lipoprotein receptor (LDLR) is connected with tumor immune response, and inhibiting LDLR can promote anti-tumor immunity. Moreover, statins can inhibit tumor metastasis by reducing the cholesterol content in the tumor cell membrane, but its curative effect needs to be further confirmed (20). Apolipoprotein E (Apo E) can mediate cholesterol metabolism and may enhance or inhibit the immune response under different circumstances, so targeting Apo E can be regarded as a direction of tumor immunotherapy (39). Besides, the metastasis of some types of tumors can be blocked by inhibiting cholesterol esterification. Extracellular fatty acids (FAs) can promote the polarization of tumor TAMs, so targeting FAs affects the development of tumors. What’s more, it is pointed out that inhibiting cholesterol efflux in TAMs can also be used as a way to treat tumors (39). However, the metabolism mechanism of cholesterol in TAMs is very complex and needs to be further explored (45).

In addition, phospholipids, a class of lipids containing phosphoric acid, have a certain influence on the TME and ultimately affect tumor proliferation and metastasis (19, 39). AA produces prostaglandin E2 (PGE2) with the help of cyclooxygenase-2 (COX2) (47). PGE2 is a tumor-related medium that can hinder the immune response and promote tumor proliferation in various ways (Y 60). TAMs can promote angiogenesis by secreting PGE2, promoting tumor development (93). Besides, PGE2 can facilitate the expression of PD-L1 in TAMs, contributing to immune escape (45). Therefore, targeting PGE2 and COX2 can hinder the expression of PD-L1 in TAMs, ultimately inhibiting immune escape. However, it is reported that the lipid metabolism regulated by different COXs in TAMs may have the opposite effects on the progression of tumors (43). It is said that cDC1 cells can enter the TME through the chemokine CCL5 secreted by NK cells, but prostaglandin E2 inhibits this process (Y. 45). Therefore, inhibiting the production of PGE2 can augment the number of cDC1 cells in TME and can cooperate with anti-PD-L1 to complete anti-tumor treatment. Suppressing the expression of COX1 or COX2 can inhibit the generation of PGE2 (86). As can be seen from the above, the COX2/PGE2 axis can be studied as a hopeful treatment (45).

CD36 is a glycoprotein that can help lipids pass through the cell membrane (19, 22). Increased expression of CD36 enhances the fatty acids oxidation in TAMs and Tregs, leading to immunosuppression (94) (**Figure 3**). Therefore, preventing these immunosuppressive cells from ingesting and metabolizing fatty acids may be a strategy worth studying to treat tumors (39). Moreover, increased expression of CD36 can also promote TAMs to ingest fatty acids and promote tumor development, so CD36 inhibitors can hinder the intake of fatty acids by TAMs and inhibit the negative impact of TAMs on immune response, ultimately achieving the purpose of inhibiting tumor progression (43). Furthermore, the high expression of CD36 can promote tumor growth and tumor metastasis by regulating the Src/PI3K/AKT signal pathway or activating the Wnt/TGF-β signal pathway (20). In addition, CD36 inhibitors can promote the submission of tumor-related antigens by DCs, decrease the amount of Treg cells, and promote the development of CD8+T cells. Using both CD36 inhibitors and anti-PD-1 could enhance the anti-tumor effects (20).

Fatty acid binding proteins (FABPs) are adipokines found in the cell membrane or cytoplasm, which can participate in the transportation of fatty acids and regulate fatty acid metabolism (95). Some studies have shown that fatty acid binding protein 4 (FABP4) can promote tumor growth, so inhibiting FABP4 can be used as a way to treat tumors (20). However, the impact of different phenotypes of FABP on tumor proliferation and development may be opposite, so it is necessary to specifically study the role of FABP to provide a basis for tumor treatment (43).

The activity of enzymes in TME connected with lipid metabolism also changes (39). ATP citric acid lyase (ACLY), fatty acid synthase (FASN), acetyl-CoA carboxylase (ACC), and acetyl-CoA (Ac CoA) are indispensable enzymes in lipid metabolism, which influence the metabolism of immune cells and tumor cells (85). ACLY decomposes cytosolic citrate in the cytoplasm into Ac CoA and oxaloacetic acid (56). ACLY inhibitors are still in their infancy, and further research is needed to provide a basis for tumor treatment (70). Ac CoA can be generated not only through ACLY but also through acetyl-CoA synthesis enzymes (ACSS). Therefore, ACSS2 inhibitors can be used to hinder the proliferation of tumors and seem to help ACLY inhibitors play a better anti-tumor role (56). ACC inhibitors combined with other treatment methods can inhibit tumor proliferation. What’s more, AMPK has been proven to be an inhibitor of ACC, and metformin can activate AMPK, so metformin is clinically used to promote anti-tumor immune response and inhibit tumor development (85). FASN is a key enzyme that regulates the *de novo* synthesis of fatty acids (20). And it catalyzes Ac CoA and malonyl-CoA to produce fatty acids (56). Inhibiting FASN can reduce the energy source of tumor cells and inhibit the role of M2 macrophages and Treg cells, eventually hindering the growth of tumors (20). But we also need to explore more deeply to obtain FASN inhibitors with better efficacy and fewer side effects to guide the clinical treatment of tumors. In addition to enzymes involved in fatty acid synthesis, enzymes related to fatty acid decomposition are also worth paying attention to (20). Carnitine palmitoyl transferase 1 (CPT1) is an enzyme involved in the beta-oxidation of fatty acids (89). It is found that inhibiting CPT1 can inhibit the role of immunosuppressive cells, such as TAMs and MDSCs, so inhibiting CPT1 can restore the immune response (20).

In addition, analyzing the signaling pathways connected with lipid metabolism reprogramming can also be used as a starting point for finding treatment methods (45). It is well-known that the AMPK/mTOR signaling pathway is related to lipid metabolism. The mTORC1 can promote the synthesis of fatty acids by regulating the activity of SREBPs, which are a class of transcription factors related to enzymes and other related molecules needed for lipid metabolism (96). As mentioned above, activating AMPK can achieve the purpose of inhibiting tumors, but it shows that AMPK may also promote the development of tumors. Therefore, AMPK agonists need to be further explored to inhibit tumor proliferation as much as possible (20). The mTOR signaling pathway is crucial for the growth of tumors, so inhibiting mTOR can play an anti-tumor role. However, it has been found that the influence of mTOR on the survival and development of immune cells is also worth paying attention to. Inhibiting mTOR may affect the role of immunosuppressive cells, such as Treg cells, hindering anti-tumor immune response (20). What’s more, it is demonstrated that the AMPK/mTOR signaling pathway influences the metabolism of CD4+T cells. Inhibiting mTOR leads to increased lipid metabolism, which is in favor of the formation and survival of Treg cells (56). Nevertheless, mTOR inhibitors also seem to inhibit the function of Treg cells, so more research is needed to find mTOR inhibitors that maximize the anti-tumor effect.

The peroxisome proliferator-activated receptor (PPAR) signaling pathway cannot be ignored (20, 97). The PPAR has been found to include peroxisome proliferator-activated receptor α (PPARα), peroxisome proliferator-activated receptor γ (PPARγ), and peroxisome proliferator-activated receptor β/δ (PPARβ/δ) (40). Among them, PPARα mainly regulates oxidative phosphorylation in glucose metabolism, while PPARγ mainly affects lipid metabolism (92). PPARγ-mediated lipid metabolism may hinder immune response and facilitate tumor growth (43). Increased expression of PPAR-γ may promote lipid synthesis and increase the activity of immune cells, ultimately enhancing immunotherapy (60). Besides, PPAR-γ agonists can inhibit tumor growth by hindering angiogenesis and facilitating the function of CD8+T cells. Furthermore, the combined use of PPAR-γ agonists and anti-PD-1 can enhance the efficacy of tumor treatment (20). Unexpectedly, PPAR-γ inhibitors can also be used to treat some tumors by inhibiting M2-like macrophages from secreting tumor-promoting cytokines (43). Therefore, it is necessary to analyze the effect of specific PPAR-γ inhibitors or agonists. Some signaling pathways also need to be studied, such as SREBP and liver X receptor (LXR) signaling pathways (20). The intake and discharge of cholesterol are regulated by SREBPs and LXRs respectively (56). It has been proved that SREBP inhibitors and LXR agonists can promote anti-tumor immune response by regulating the role of immune cells (56). Interestingly, using both LXR agonists and PPAR inhibitors seems to enhance the anti-tumor effect (20).

There are many points worth paying attention to in normal lipid metabolism and lipid metabolism reprogramming. Analyzing them as deeply as possible may provide some basis for tumor treatment. In tumor tissue, the anabolic and catabolism of lipids are unbalanced, leading to lipid accumulation, which eventually affects the therapeutic effect and prognosis (98). It is not difficult to find that targeting transport proteins connected with fatty acid transport can inhibit the growth of tumors, but there are many ways to transport fatty acids. The treatment methods to block these routes need to be further studied as much as possible (22). It is worth noting that specific studies should be carried out on different stages of lipid metabolism to obtain treatment methods that can help improve the effect of immunotherapy (20). What’s more, in different TMEs, the mechanism of lipid metabolism may be different, so different tumors may require specific treatments (45).

Obesity is one of the manifestations of lipid metabolism disorders. It has been found that excessive lipids may hinder the role of NK cells (60). And if the role of NK cells is impaired, the risk of tumors in obese people may increase. Besides, diverse degrees of obesity may have disparate effects on lipid metabolism (60). As the number of obese people increases year by year, it is particularly vital to analyze the features of lipid metabolism in immune cells and tumor cells. Whether obesity can lead to a bad prognosis of tumors is also worth an in-depth study (58).

#### 4.2.3 Therapies related to amino acid metabolism

Of Note, tumor cells need more amino acids than normal cells and have stronger amino acid metabolism. Therefore, targeting amino acid metabolism can hinder the function of tumor cells without excessive damage to normal cells (17). Amino acid metabolism can affect the role of immune cells and the production of immune factors, which has a significant effect on tumor immunity (17). Targeting amino acid metabolism can restore tumor immune response, so it is worth studying deeply as a tumor treatment strategy. Immune checkpoint receptors, including PD-1 and CTLA4, limit the role of T cells by hindering T cells from ingesting and catabolizing amino acids (17). Hence, blocking immune checkpoints can promote amino acid metabolism in T cells (17).

Amino acid transporters are of significance in tumor proliferation and metastasis. It is reported that one of the ways that amino acid transporters affect tumor growth is to regulate the activity of mTORC1, for example, recombinant large neutral amino acid transporter 1 (LAT1) can increase the activity of mTORC1 to facilitate tumor growth and metastasis (17). It has shown that LAT1 can activate mTORC1 through the Ragulator-Rag complex and help to transmit VEGF-A signals through mTORC1 and promote angiogenesis (17). When the amount of LAT1 becomes less, the activity of mTORC1 decreases, and this is not good for tumor development (17). Hence, amino acid transporters can be used as targets for tumor treatment. However, there is a great deal of amino acid transport proteins in TME, thus inhibiting one amino acid transport protein may have an impact on other amino acid transport proteins (17).

Indoleamine 2,3-dioxygenase 1 (IDO1), indoleamine 2,3-dioxygenase 2 (IDO2) and Tryptophan-2,3-dioxygenase (TDO) are three enzymes in the tryptophan metabolism, and IDO1 is highly expressed (15). IDO1 promotes tumor development through immunosuppression, while TDO is connected with tumor proliferation and metastasis (98). Therefore, it is necessary to deeply analyze the role of IDO and its relevance to TDO. IDO, produced by tumor cells, TAMs and Tregs in the TME, decomposes tryptophan and generates specific metabolites (5) (**Figure 3**). IDO can inhibit the role of immune cells, such as effector T cells, and promote the formation of an immunosuppressive microenvironment (5). It has proved that dendritic cells expressing IDO can facilitate the production and proliferation of Treg cells, ultimately hindering tumor immunity (Xiao-han 99). And it can be seen that targeting IDO1, which is considered one of the meaningful strategies for treating tumors, can inhibit tumor development by restoring tumor immunity (83). There are also difficulties in the study of IDO/TDO inhibitors. Tumor cells may produce kynurenine (KYN), one of the metabolites of tryptophan, by using IDO or TDO alone, or by using IDO and TDO at the same time. Therefore, it is very important to identify the enzymes that produce KYN and select specific inhibitors for tumor treatment. For example, Epacadostat can inhibit the secretion of KYN by tumor cells that mainly express IDO1, but it hardly affects the tumor cells that mainly express TDO (83). Of course, the dose of inhibitors is also a factor that cannot be ignored (99). However, in addition to using IDO1 inhibitors alone, using both IDO1 inhibitors and ICB is also worth studying carefully. Furthermore, whether the use of both IDO inhibitors and PD-1/PD-L1 inhibitors is better than that of a certain inhibitor alone is worth further study (99). For instance, the IDO1 inhibitor, LY3381916, combined with the PD-L1 inhibitor, can minimize the activity of IDO1, eventually increasing the number of T cells and promoting anti-tumor immunity (15).

Glutamine can activate mTORC1 to promote tumor cell proliferation (5). Glutamine antagonists, such as JHU083, can inhibit glutamine metabolism in tumor cells and promote the formation of the TME beneficial for the survival of immune cells, ultimately achieving the purpose of treating tumors (5). Moreover, the simultaneous use of glutamine antagonists and anti-PD-1 antibodies can enhance the function of T cells, and its therapeutic effect is better than the use of antiPD-1 antibodies alone (5). Glutaminase (GLS), highly expressed in tumor cells, is an enzyme that breaks down glutamine into glutamate. Therefore, inhibiting glutamine enzymes may be used as a way to treat tumors (17). Besides, it is found that GLS antagonists, such as CB-839, can increase the content of Gln in TME by inhibiting the utilization of Gln by tumor cells. When Gln in TME increases, some signaling pathways can be activated to strengthen the anti-tumor activity of NK cells (17).

The growth of multiple T cells is connected with the amount of amino acids. Promoting the inflow of amino acids is conducive to the growth of T cells and eventually affects the anti-tumor immune response (75). Besides, it is indicated that the function of effector T cells can be improved by supplementing amino acids, and anti-tumor immunity can be enhanced when anti-PD-L1 antibody therapy is performed at the same time (5). Supplementing arginine or targeting enzymes related to the arginine metabolism probably enhances the role of T cells and reduces immunosuppression (15). Inhibiting the catabolism pathways of tryptophan and arginine hinders the function of Tregs in anti-tumor immunity, so these pathways could be taken as targets for tumor immunotherapy (89).

To sum up, targeting the amino acid transporters and metabolic enzymes related to amino acid metabolism reprogramming in TME is a hopeful strategy for tumor treatment, which can enhance anti-tumor immunity and inhibit the proliferation and metastasis of tumors with immune checkpoint inhibitors (17). Apparently, amino acid metabolism is important for tumor proliferation and metastasis. Of course, the connection between amino acid metabolism and other cells, such as vascular endothelial cells, in TME, is also worth continuing to study to have a deeper understanding of tumors and develop new promising treatment strategies (5). Although the current understanding of amino acid metabolism reprogramming in TME is limited, targeting amino acid metabolism is still a strategy worth studying to enhance anti-tumor immunity. Hence, more efforts are needed to study the significance of amino acid metabolism for tumor immunotherapy (15).

## 5 Discussion

Tumor cells and immune cells are two types of cells that cannot be ignored in TME, and their metabolism can affect the progression of tumors. Tumor cells change their metabolic patterns, namely metabolism reprogramming, to adapt to the TME and escape immune surveillance, ultimately facilitating the rapid proliferation and metastasis of tumors. The immune system includes anti-tumor immune cells, such as effector T (Teff) cells and M1 macrophages, as well as immunosuppressive cells that promote tumor development, such as Treg cells and M2 macrophages. In theory, anti-tumor immune cells can inhibit the proliferation of tumors by recognizing and destroying tumor cells to maintain the homeostasis of the body. However, tumor cells in TME can hinder the function of anti-tumor immune cells and promote the function of immunosuppressive cells through metabolism reprogramming, eventually forming an immunosuppressive microenvironment and promoting tumor development. Therefore, analyzing the metabolism reprogramming in TME and the impact of metabolism reprogramming on immune cells may be an important approach for immunotherapy.

Tumor cells in different TMEs have diverse metabolic characteristics. The metabolism of tumor cells could be compensated, so the effect of antitumor treatment through a single target may be short-lived. For enhancing the efficacy of anti-tumor treatment, implementing multiple therapies at the same time may be a more comprehensive treatment. Future research should distinguish more clearly the same and different points between the metabolism of tumor cells and immune cells to analyze targeted treatment strategies so that targeting metabolism can better play an anti-tumor role together with immunotherapy.

Immunotherapy mainly includes specific immunotherapy and nonspecific immunotherapy (17). Tumor immunotherapy, such as blocking immune checkpoints, has been widely used in clinics. However, there are still some diseases that cannot be solved with existing immunotherapy (100). Furthermore, at diverse stages, the metabolic modes of tumor cells and immune cells may be disparate (60). However, the types and quantities of immune cells contained in different TME are different. Immunotherapy is more effective in tumors with more immune cells. It is demonstrated that autophagy, as the self-protection mechanism of cells, has a non-negligible effect on tumor cells and immune cells. Inhibiting autophagy probably be beneficial for anti-tumor immune response, so it may be a new immunotherapy method (20). Genes connected with metabolism possibly become targets for immunotherapy (101).

Although TME and tumor immunotherapy are still being explored, we can predict that immunotherapy can continue being expanded and developed with the development of science and technology to promote the treatment and prognosis of tumors.

## Author contributions

YL: Writing – original draft. HS: Writing – original draft. YH: Supervision, Writing – review & editing. XQ: Supervision, Writing – review & editing.

## References

[1] Fukushi A Kim HD Chang YC Kim CH. Revisited metabolic control and reprogramming cancers by means of the warburg effect in tumor cells. Int J Mol Sci (2022) 23(17):10037. doi: 10.3390/ijms231710037 36077431 PMC9456516

[2] Li X Xu W. CD147−mediated reprogrammed glycolytic metabolism potentially induces immune escape in the tumor microenvironment (Review). Oncol Rep (2019) 41(5):2945–56. doi: 10.3892/or.2019.7041 30864716

[3] Kooshki L Mahdavi P Fakhri S Akkol EK Khan H. Targeting lactate metabolism and glycolytic pathways in the tumor microenvironment by natural products: A promising strategy in combating cancer. BioFactors (2022) 48(2):359–83. doi: 10.1002/biof.1799 34724274

[4] Wu S Zhang H Gao C Chen J Li H Meng Z. Hyperglycemia enhances immunosuppression and aerobic glycolysis of pancreatic cancer through upregulating bmi1-UPF1-HK2 pathway. Cell Mol Gastroenterol Hepatol (2022) 14(5):1146–65. doi: 10.1016/j.jcmgh.2022.07.008 PMC9606831 35863742

[5] Qiu H Shao N Liu J Zhao J Chen C Li Q. Amino acid metabolism in tumor: new shine in the fog? Clin Nutr (2023) 42(8):1521–305. doi: 10.1016/j.clnu.2023.06.011 37321900

[6] Kong J Yu G Si W Li G Chai J Liu Y. Identification of a glycolysis-related gene signature for predicting prognosis in patients with hepatocellular carcinoma. BMC Cancer (2022) 22(1):142. doi: 10.1186/s12885-022-09209-9 35123420 PMC8817563

[7] Yang J Liu DJ Zheng JH He RZ Xu DP Yang MW. IRAK2-NF-κB signaling promotes glycolysisDependent tumor growth in pancreatic cancer. Cell Oncol (Dordr) (2022) 45(3):367–79. doi: 10.1007/s13402-022-00670-z PMC12978042 35486320

[8] Xiang H Yang R Tu J Xi Y Yang S Lv L. Metabolic reprogramming of immune cells in pancreatic cancer progression. Biomed Pharmacother (2023) 157(January):113992. doi: 10.1016/j.biopha.2022.113992 36395610

[9] Li Y Zhao L Li XF. Hypoxia and the tumor microenvironment. Technol Cancer Res Treat (2021) 20(December):15330338211036304. doi: 10.1177/15330338211036304 34350796 PMC8358492

[10] Sun L Suo C Li S-t Zhang H Gao P. Metabolic reprogramming for cancer cells and their microenvironment: Beyond the Warburg Effect. Biochim Biophys Acta (BBA) - Rev Cancer Cancer Metab (2018) 1870 (1):51–66. doi: 10.1016/j.bbcan.2018.06.005 29959989

[11] Lin Y Xiao Y Liu S Hong L Shao L Wu J. Role of a lipid metabolism-related lncRNA signature in risk stratification and immune microenvironment for colon cancer. BMC Med Genomics (2022) 15(1):221. doi: 10.1186/s12920-022-01369-8 36280825 PMC9590147

[12] Zhu L Zhu X Wu Y. Effects of glucose metabolism, lipid metabolism, and glutamine metabolism on tumor microenvironment and clinical implications. Biomolecules (2022) 12(4):580. doi: 10.3390/biom12040580 35454171 PMC9028125

[13] Reina-Campos M Moscat J Diaz-Meco M. Metabolism shapes the tumor microenvironment. Curr Opin Cell Biol (2017) 48(October):47–53. doi: 10.1016/j.ceb.2017.05.006 28605656 PMC5650101

[14] Aung A Kumar V Theprungsirikul J Davey SK Varghese S. An engineered tumor-on-a-chip device with breast cancer-immune cell interactions for assessing T-cell recruitment. Cancer Res (2020) 80(2):263–75. doi: 10.1158/00085472.Can-19-0342 PMC8545579 31744818

[15] Yu M Zhang S. Influenced tumor microenvironment and tumor immunity by amino acids. Front Immunol (2023) 14:1118448. doi: 10.3389/fimmu.2023.1118448 36798123 PMC9927402

[16] Zhang Y Hu H Liu W Yan SM Li Y Tan L. Amino acids and ragD potentiate mTORC1 activation in CD8(+) T cells to confer antitumor immunity. J Immunother Cancer (2021) 9(4):e002137. doi: 10.1136/jitc-2020-002137 33883257 PMC8061841

[17] Wang D Wan X. Progress in research on the role of amino acid metabolic reprogramming in tumour therapy: A review. Biomed Pharmacother (2022) 156(December):113923. doi: 10.1016/j.biopha.2022.113923 36411616

[18] Li S Fang Y. MS4A1 as a potential independent prognostic factor of breast cancer related to lipid metabolism and immune microenvironment based on TCGA database analysis. Med Sci Monit (2022) 28(January):e934597. doi: 10.12659/msm.934597 35091527 PMC8809038

[19] An D Zhai D Wan C Yang K. The role of lipid metabolism in cancer radioresistance. Clin Transl Oncol (2023) 25(8):2332–49. doi: 10.1007/s12094-023-03134-4 37079212

[20] Yang K Wang X Song C He Z Wang R Xu Y. The role of lipid metabolic reprogramming in tumor microenvironment. Theranostics (2023) 13(6):1774–808. doi: 10.7150/thno.82920 PMC10091885 37064872

[21] Kolb D Kolishetti N Surnar B Sarkar S Guin S Shah AS. Metabolic modulation of the tumor microenvironment leads to multiple checkpoint inhibition and immune cell infiltration. ACS Nano (2020) 14(9):11055–66. doi: 10.1021/acsnano.9b10037 32706241

[22] Corn KC Windham MA Rafat M. Lipids in the tumor microenvironment: from cancer progression to treatment. Prog Lipid Res (2020) 80(November):101055. doi: 10.1016/j.plipres.2020.101055 32791170 PMC7674189

[23] Hao Y Li D Xu Y Ouyang J Wang Y Zhang Y. Investigation of lipid metabolism dysregulation and the effects on immune microenvironments in pan-cancer using multiple omics data. BMC Bioinf (2019) 20(Suppl 7):195. doi: 10.1186/s12859-019-2734-4 PMC6509864 31074374

[24] Gu X Wei S Li Z Xu H. Machine learning reveals two heterogeneous subtypes to assist immune therapy based on lipid metabolism in lung adenocarcinoma. Front Immunol (2022) 13:1022149. doi: 10.3389/fimmu.2022.1022149 36238302 PMC9551187

[25] Yang Y Li Y Qi R Zhang L. Development and validation of a combined glycolysis and immune prognostic model for melanoma. Front Immunol (2021) 12:711145. doi: 10.3389/fimmu.2021.711145 34659201 PMC8517401

[26] Ossoli A Wolska A Remaley AT Gomaraschi M. High-density lipoproteins: A promising tool against cancer. Biochim Biophys Acta (BBA) - Mol Cell Biol Lipids (2022) 1867(1):159068. doi: 10.1016/j.bbalip.2021.159068 34653581

[27] Xu K Xia P Liu P Zhang X. A six lipid metabolism related gene signature for predicting the prognosis of hepatocellular carcinoma. Sci Rep (2022) 12(1):20781. doi: 10.1038/s41598-022-25356-2 36456877 PMC9715694

[28] Chen YJ Guo X Liu ML Yu YY Cui YH Shen XZ. Interaction between glycolysis−cholesterol synthesis axis and tumor microenvironment reveal that gamma-glutamyl hydrolase suppresses glycolysis in colon cancer. Front Immunol (2022) 13:979521. doi: 10.3389/fimmu.2022.979521 36569910 PMC9767965

[29] Yuan Y Song J Wu Q. Aberrant gene expression pattern in the glycolysis-cholesterol synthesis axis is linked with immune infiltration and prognosis in prostate cancer: A bioinformatics analysis. Med (Baltimore) (2022) 101(43):e31416. doi: 10.1097/md.0000000000031416 PMC9622640 36316896

[30] Chen F Chen J Yang L Liu J Zhang X Zhang Y. Extracellular vesicle-packaged HIF-1α-stabilizing lncRNA from tumour-associated macrophages regulates aerobic glycolysis of breast cancer cells. Nat Cell Biol (2019) 21(4):498–510. doi: 10.1038/s41556-019-0299-0 30936474

[31] Cohen IJ Pareja F Socci ND Shen R Doane AS Schwartz J. Increased tumor glycolysis is associated with decreased immune infiltration across human solid tumors. Front Immunol (2022) 13:880959. doi: 10.3389/fimmu.2022.880959 36505421 PMC9731115

[32] Xu W Weng J Xu M Zhou Q Liu S Hu Z. Functions of key enzymes of glycolytic metabolism in tumor microenvironment. Cell Reprogram (2023) 25(3):91–8. doi: 10.1089/cell.2023.0010 37172278

[33] Xia KG Wang CM Shen DY Song XY Mu XY Zhou JW. LncRNA NEAT1Associated aerobic glycolysis blunts tumor immunosurveillance by T cells in prostate cancer. Neoplasma (2022) 69(3):594–602. doi: 10.4149/neo_2022_211021N1497 35263995

[34] Chelakkot C Chelakkot VS Shin Y Song K. Modulating glycolysis to improve cancer therapy. Int J Mol Sci (2023) 24(3):2606. doi: 10.3390/ijms24032606 36768924 PMC9916680

[35] Webb BA Chimenti M Jacobson MP Barber DL. Dysregulated pH: a perfect storm for cancer progression. Nat Rev Cancer (2011) 11(9):671–7. doi: 10.1038/nrc3110 21833026

[36] Bi J Bi F Pan X Yang Q. Establishment of a novel glycolysis-related prognostic gene signature for ovarian cancer and its relationships with immune infiltration of the tumor microenvironment. J Transl Med (2021) 19(1):382. doi: 10.1186/s12967021-03057-0 34496868 PMC8425093

[37] Da Q Huang L Huang C Chen Z Jiang Z Huang F. Glycolytic regulatory enzyme PFKFB3 as a prognostic and tumor microenvironment biomarker in human cancers. Aging (Albany NY) (2023) 15(10):4533–59. doi: 10.18632/aging.204758 PMC10258027 37253634

[38] Ma Y Zhang S Jin Z Shi M. LipidMediated regulation of the cancer-immune crosstalk. Pharmacol Res (2020) 161(November):105131. doi: 10.1016/j.phrs.2020.105131 32810628

[39] Ma K Zhang L. Overview: lipid metabolism in the tumor microenvironment. Adv Exp Med Biol (2021) 1316:41–7. doi: 10.1007/978-981-33-6785-2_3 33740242

[40] Zhong J Guo J Zhang X Feng S Di W Wang Y. The remodeling roles of lipid metabolism in colorectal cancer cells and immune microenvironment. Oncol Res (2022) 30(5):231–42. doi: 10.32604/or.2022.027900 PMC10207963 37305350

[41] Li J Zhang S Chen S Yuan Y Zuo M Li T. Lipid metabolism-related gene signature predicts prognosis and depicts tumor microenvironment immune landscape in gliomas. Front Immunol (2023) 14:1021678. doi: 10.3389/fimmu.2023.1021678 36860853 PMC9968762

[42] Shen L Huang H Li J Chen W Yao Y Hu J. Exploration of prognosis and immunometabolism landscapes in ER+ Breast cancer based on a novel lipid metabolism-related signature. Front Immunol (2023) 14:1199465. doi: 10.3389/fimmu.2023.1199465 37469520 PMC10352658

[43] Qiao X Hu Z Xiong F Yang Y Peng C Wang D. Lipid metabolism reprogramming in tumor-associated macrophages and implications for therapy. Lipids Health Dis (2023) 22(1):45. doi: 10.1186/s12944-023-01807-1 37004014 PMC10064535

[44] Liu L Mo M Chen X Chao D Zhang Y Chen X. Targeting inhibition of prognosis-related lipid metabolism genes including CYP19A1 enhances immunotherapeutic response in colon cancer. J Exp Clin Cancer Res (2023) 42(1):85. doi: 10.1186/s13046-023-02647-8 37055842 PMC10100168

[45] Xiang Y Miao H. Lipid metabolism in tumor-associated macrophages. Adv Exp Med Biol (2021) 1316:87–101. doi: 10.1007/978981-33-6785-2_6 33740245

[46] Jiang A Chen X Zheng H Liu N Ding Q Li Y. Lipid metabolism-related gene prognostic index (LMRGPI) reveals distinct prognosis and treatment patterns for patients with early-stage pulmonary adenocarcinoma. Int J Med Sci (2022) 19(4):711–28. doi: 10.7150/ijms.71267 PMC9108406 35582412

[47] Dai J Li Q Quan J Webb G Liu J Gao K. Construction of a lipid metabolism-related and immune-associated prognostic score for gastric cancer. BMC Med Genomics (2023) 16(1):93. doi: 10.1186/s12920-023-01515-w 37138287 PMC10158005

[48] Wang R Liu Z Fan Z Zhan H. Lipid metabolism reprogramming of CD8(+) T cell and therapeutic implications in cancer. Cancer Lett (2023) 567(July):216267. doi: 10.1016/j.canlet.2023.216267 37315709

[49] Offer S Menard JA Pérez JE de Oliveira KG Indira Chandran V Johansson MC. Extracellular lipid loading augments hypoxic paracrine signaling and promotes glioma angiogenesis and macrophage infiltration. J Exp Clin Cancer Res (2019) 38(1):241. doi: 10.1186/s13046-019-1228-6 31174567 PMC6556032

[50] Yu Z Zhou Y Li Y Dong Z. Integration of clinical and spatial data to explore lipid metabolism-related genes for predicting prognosis and immune microenvironment in gliomas. Funct Integr Genomics (2023) 23(2):82. doi: 10.1007/s10142-02301010-6 36929451

[51] Xiong Y Si Y Feng Y Zhuo S Cui B Zhang Z. Prognostic value of lipid metabolism-related genes in head and neck squamous cell carcinoma. Immun Inflammation Dis (2021) 9(1):196–209. doi: 10.1002/iid3.379 PMC7860527 33277966

[52] Li Z Jin C Lu X Zhang Y Zhang Y Wen J. Studying the mechanism underlying lipid metabolism in osteosarcoma based on transcriptomic RNA sequencing and single-cell data. J Gene Med (2023) 25(6):e3491. doi: 10.1002/jgm.3491 36847293

[53] Chen J Ye J Lai R. A lipid metabolism-related gene signature reveals dynamic immune infiltration of the colorectal adenoma-carcinoma sequence. Lipids Health Dis (2023) 22(1):92. doi: 10.1186/s12944-023-01866-4 37403152 PMC10318759

[54] Chen Y Yuan H Yu Q Pang J Sheng M Tang W. Bioinformatics analysis and structure of gastric cancer prognosis model based on lipid metabolism and immune microenvironment. Genes (Basel) (2022) 13(9):1581. doi: 10.3390/genes13091581 36140749 PMC9498347

[55] Mylonis I Simos G Paraskeva E. Hypoxia-inducible factors and the regulation of lipid metabolism. Cells (2019) 8(3):214. doi: 10.3390/cells8030214 30832409 PMC6468845

[56] He S Cai T Yuan J Zheng X Yang W. Lipid metabolism in tumor-infiltrating T cells. Adv Exp Med Biol (2021) 1316:149–67. doi: 10.1007/978-981-33-6785-2_10 33740249

[57] Liu X Hartman CL Li L Albert CJ Si F Gao A. Reprogramming lipid metabolism prevents effector T cell senescence and enhances tumor immunotherapy. Sci Transl Med (2021) 13(587):eaaz6314. doi: 10.1126/scitranslmed.aaz6314 33790024 PMC12040281

[58] Prendeville H Lynch L. Diet, lipids, and antitumor immunity. Cell Mol Immunol (2022) 19(3):432–44. doi: 10.1038/s41423021-00781-x PMC8891265 34983949

[59] Fultang L Gamble LD Gneo L Berry AM Egan SA De Bie F. Macrophage-derived IL1β and TNFα Regulate arginine metabolism in neuroblastoma. Cancer Res (2019) 79(3):611–24. doi: 10.1158/0008-5472.Can-18-2139 PMC6420118 30545920

[60] Chen Y Sui M. Lipid metabolism in tumor-associated natural killer cells. Adv Exp Med Biol (2021) 1316:71–85. doi: 10.1007/978-981-33-6785-2_5 33740244

[61] Wang S Liu R Yu Q Dong L Bi Y Liu G. Metabolic reprogramming of macrophages during infections and cancer. Cancer Lett (2019) 452(June):14–22. doi: 10.1016/j.canlet.2019.03.015 30905817

[62] Li W Xu M Li Y Huang Z Zhou J Zhao Q. Comprehensive analysis of the association between tumor glycolysis and immune/inflammation function in breast cancer. J Transl Med (2020) 18(1):92. doi: 10.1186/s12967-02002267-2 32070368 PMC7029444

[63] Yang F Wan F. Lipid metabolism in tumor-associated B cells. Adv Exp Med Biol (2021) 1316:133–47. doi: 10.1007/978-981-336785-2_9 33740248

[64] Jung JG Le A. Metabolism of immune cells in the tumor microenvironment. Adv Exp Med Biol (2021) 1311:173–85. doi: 10.1007/978-3-030-65768-0_13 PMC9703210 34014543

[65] Arabzadeh A Quail DF. Myosin II in cancer cells shapes the immune microenvironment. Trends Mol Med (2019) 25(4):257–59. doi: 10.1016/j.molmed.2019.02.011 30871808

[66] Zheng J Xu W Liu W Tang H Lu J Yu K. Traditional chinese medicine bu-shen-jian-pi-fang attenuates glycolysis and immune escape in clear cell renal cell carcinoma: results based on network pharmacology. Biosci Rep (2021) 41(6):BSR20204421. doi: 10.1042/bsr20204421 34002799 PMC8202066

[67] Li X Zhang Y Ma W Fu Q Liu J Yin G. Enhanced glucose metabolism mediated by CD147 contributes to immunosuppression in hepatocellular carcinoma. Cancer Immunol Immunother (2020) 69(4):535–48. doi: 10.1007/s00262-01902457-y PMC11027868 31965268

[68] Li Y Song Z Han Q Zhao H Pan Z Lei Z. Targeted inhibition of STAT3 induces immunogenic cell death of hepatocellular carcinoma cells *via* glycolysis. Mol Oncol (2022) 16(15):2861–80. doi: 10.1002/1878-0261.13263 PMC9348600 35665592

[69] Yu W Lei Q Yang L Qin G Liu S Wang D. Contradictory roles of lipid metabolism in immune response within the tumor microenvironment. J Hematol Oncol (2021) 14(1):187. doi: 10.1186/s13045-021-01200-4 34742349 PMC8572421

[70] Zipinotti Dos Santos D de Souza JC Pimenta TM da Silva Martins B Junior RSR Butzene SMS. The impact of lipid metabolism on breast cancer: A review about its role in tumorigenesis and immune escape. Cell Commun Signal (2023) 21(1):161. doi: 10.1186/s12964-023-01178-1 37370164 PMC10304265

[71] Flerin NC Pinioti S Menga A Castegna A Mazzone M. Impact of immunometabolism on cancer metastasis: A focus on T cells and macrophages. Cold Spring Harb Perspect Med (2020) 10(9):a037044. doi: 10.1101/cshperspect.a037044 31615868 PMC7461771

[72] Huang Y. Targeting glycolysis for cancer therapy using drug delivery systems. J Controlled Release (2023) 353(January):650–62. doi: 10.1016/j.jconrel.2022.12.003 36493949

[73] Zeng L Liang L Fang X Xiang S Dai C Zheng T. Glycolysis induces th2 cell infiltration and significantly affects prognosis and immunotherapy response to lung adenocarcinoma. Funct Integr Genomics (2023) 23(3):221. doi: 10.1007/s10142-023-01155-4 37400733

[74] Zhao X Lian X Xie J Liu G. Accumulated cholesterol protects tumours from elevated lipid peroxidation in the microenvironment. Redox Biol (2023) 62(June):102678. doi: 10.1016/j.redox.2023.102678 36940607 PMC10036943

[75] Panetti S McJannett N Fultang L Booth S Gneo L Scarpa U. Engineering amino acid uptake or catabolism promotes CAR T-cell adaption to the tumor environment. Blood Adv (2023) 7(9):1754–61. doi: 10.1182/bloodadvances.2022008272 PMC10182289 36521029

[76] Luo Q Zheng N Jiang L Wang T Zhang P Liu Y. Lipid accumulation in macrophages confers protumorigenic polarization and immunity in gastric cancer. Cancer Sci (2020) 111(11):4000–11. doi: 10.1111/cas.14616 PMC7648032 32798273

[77] Bao D Zhao J Zhou X Yang Q Chen Y Zhu J. Mitochondrial fission-induced mtDNA stress promotes tumor-associated macrophage infiltration and HCC progression. Oncogene (2019) 38(25):5007–20. doi: 10.1038/s41388-019-0772-z PMC6755992 30894684

[78] Bohn T Rapp S Luther N Klein M Bruehl TJ Kojima N. Tumor immunoevasion *via* acidosisDependent induction of regulatory tumor-associated macrophages. Nat Immunol (2018) 19(12):1319–29. doi: 10.1038/s41590-018-0226-8 30397348

[79] Li M Yang Y Xiong L Jiang P Wang J Li C. Metabolism, metabolites, and macrophages in cancer. J Hematol Oncol (2023) 16(1):80. doi: 10.1186/s13045-023-01478-6 37491279 PMC10367370

[80] Cao J Zeng F Liao S Cao L Zhou Y. Effects of glycolysis on the polarization and function of tumor−associated macrophages (Review). Int J Oncol (2023) 62(6):70. doi: 10.3892/ijo.2023.5518 37144503 PMC10198715

[81] Zang X Zhang X Hu H Qiao M Zhao X Deng Y. Targeted delivery of zoledronate to tumor-associated macrophages for cancer immunotherapy. Mol Pharm (2019) 16(5):2249–58. doi: 10.1021/acs.molpharmaceut.9b00261 30969779

[82] Weigert A Strack E Snodgrass RG Brüne B. mPGES-1 and ALOX5/-15 in tumor-associated macrophages. Cancer Metastasis Rev (2018) 37(2–3):317–34. doi: 10.1007/s10555-018-9731-3 29808459

[83] Zhan Y Qiao W Yi B Yang X Li M Sun L. Dual role of pseudogene TMEM198B in promoting lipid metabolism and immune escape of glioma cells. Oncogene (2022) 41(40):4512–23. doi: 10.1038/s41388-022-02445-0 36038663

[84] Ye J Yang Y Dong W Gao Y Meng Y Wang H. Drug-free mannosylated liposomes inhibit tumor growth by promoting the polarization of tumor-associated macrophages. Int J Nanomed (2019) 14:3203–20. doi: 10.2147/ijn.S207589 PMC6509939 31118632

[85] Kobayashi T Lam PY Jiang H Bednarska K Gloury R Murigneux V. Increased lipid metabolism impairs NK cell function and mediates adaptation to the lymphoma environment. Blood (2020) 136(26):3004–17. doi: 10.1182/blood.2020005602 32818230

[86] Yenyuwadee S Aliazis K Wang Qi Christofides A Shah R Patsoukis N. Immune cellular components and signaling pathways in the tumor microenvironment. Semin Cancer Biol (2022) 86(November):187–201. doi: 10.1016/j.semcancer.2022.08.004 35985559 PMC10735089

[87] Hicks KC Tyurina YY Kagan VE Gabrilovich DI. Myeloid cell-derived oxidized lipids and regulation of the tumor microenvironment. Cancer Res (2022) 82(2):187–94. doi: 10.1158/0008-5472.Can-21-3054 PMC8770601 34764204

[88] Qin H Chen Y. Lipid metabolism and tumor antigen presentation. Adv Exp Med Biol (2021) 1316:169–89. doi: 10.1007/978981-33-6785-2_11 33740250

[89] Lemos H Huang L Prendergast GC Mellor AL. Immune control by amino acid catabolism during tumorigenesis and therapy. Nat Rev Cancer (2019) 19(3):162–75. doi: 10.1038/s41568019-0106-z 30696923

[90] Takahashi H Kawabata-Iwakawa R Ida S Mito I Tada H Chikamatsu K. Upregulated glycolysis correlates with tumor progression and immune evasion in head and neck squamous cell carcinoma. Sci Rep (2021) 11(1):17789. doi: 10.1038/s41598-02197292-6 34493792 PMC8423753

[91] Ho KH Huang TW Shih CM Lee YT Liu AJ Chen PH. Glycolysis-associated lncRNAs identify a subgroup of cancer patients with poor prognoses and a highInfiltration immune microenvironment. BMC Med (2021) 19(1):59. doi: 10.1186/s12916-021-01925-6 33627136 PMC7905662

[92] Çolakoğlu M Tunçer S Banerjee S. Emerging cellular functions of the lipid metabolizing enzyme 15-lipoxygenase-1. Cell Prolif (2018) 51(5):e12472. doi: 10.1111/cpr.12472 30062726 PMC6528901

[93] Chang X Xing P. Identification of a novel lipid metabolism-related gene signature within the tumour immune microenvironment for breast cancer. Lipids Health Dis (2022) 21(1):43. doi: 10.1186/s12944-022-01651-9 35562758 PMC9103058

[94] Yan D Adeshakin AO Xu M Afolabi LO Zhang G Chen YH. Lipid metabolic pathways confer the immunosuppressive function of myeloid-derived suppressor cells in tumor. Front Immunol (2019) 10:1399. doi: 10.3389/fimmu.2019.01399 31275326 PMC6593140

[95] Hu BO Lin J-Z Yang X-B Sang X-T. Aberrant lipid metabolism in hepatocellular carcinoma cells as well as immune microenvironment: A review. Cell Proliferation (2020) 53(3):e127725. doi: 10.1111/cpr.12772 PMC7106960 32003505

[96] Di Conza G Tsai CH Gallart-Ayala H Yu YR Franco F Zaffalon L. Tumor-induced reshuffling of lipid composition on the endoplasmic reticulum membrane sustains macrophage survival and pro-tumorigenic activity. Nat Immunol (2021) 22(11):1403–15. doi: 10.1038/s41590-021-01047-4 PMC7611917 34686867

[97] Zeng W Yin X Jiang Y Jin L Liang W. PPARα at the crossroad of metabolic-immune regulation in cancer. FEBS J (2022) 289(24):7726–39. doi: 10.1111/febs.16181 34480827

[98] Trézéguet V Fatrouni H Merched AJ. Immuno-metabolic modulation of liver oncogenesis by the tryptophan metabolism. Cells (2021) 10(12):3469. doi: 10.3390/cells10123469 34943977 PMC8700200

[99] Liu X-h Zhai X-y. Role of tryptophan metabolism in cancers and therapeutic implications. Biochimie (2021) 182(March):131–39. doi: 10.1016/j.biochi.2021.01.005 33460767

[100] Jiang Z Liu Z Li M Chen C Wang X. Increased glycolysis correlates with elevated immune activity in tumor immune microenvironment. EBioMedicine (2019) 42(April):431–42. doi: 10.1016/j.ebiom.2019.03.068 PMC6491961 30935888

[101] Guo Z Liang J. Characterization of a lipid droplet and endoplasmic reticulum stress related gene risk signature to evaluate the clinical and biological value in hepatocellular carcinoma. Lipids Health Dis (2022) 21(1):146. doi: 10.1186/s12944022-01759-y 36581927 PMC9798721

